# Measuring navigational and digital health literacy in individuals with long-term conditions: latent trait analyses using Rasch modelling

**DOI:** 10.3389/fpubh.2026.1751340

**Published:** 2026-05-04

**Authors:** Mette Haaland, Hanne S. Finbråten, Sølvi Helseth, Christopher Le, Helge Skirbekk, Øystein Guttersrud

**Affiliations:** 1 Faculty of Health Sciences, Department of Nursing and Health Promotion, Oslo Metropolitan University, Oslo, Norway; 2 Norwegian Center for Health Literacy and Coping, Health Literacy Section, Department of Education and Competence Development, Oslo University Hospital, Oslo, Norway; 3 Faculty of Social and Health Sciences, Department of Health and Nursing Sciences, University of Inland Norway, Elverum, Norway; 4 The Norwegian Directorate of Health, Oslo, Norway; 5 Faculty of Mathematics and Natural Sciences, Norwegian Centre for Science Education, University of Oslo, Oslo, Norway

**Keywords:** digital health literacy, health literacy, health literacy assessment, long-term conditions, navigational health literacy, non-communicable diseases

## Abstract

**Background:**

Mapping health literacy (HL) enables health information and treatment to align with individuals’ preferences and potentially makes healthcare truly person-centered. Without valid and reliable measurement tools however, we risk misreading what people actually need. This study aimed to evaluate two measurement scales developed to assess navigational and digital HL in a population sample of individuals with long-term conditions (LTCs).

**Methods:**

This secondary study used cross-sectional data from the large-scale Health Literacy Population Survey 2019–2021. The two scales were evaluated in a national population (≥ 18 years) with self-reported long-term conditions (duration ≥ 6 months). A latent trait analysis using the unidimensional Rasch partial credit model (PCM) for polytomous responses was performed to evaluate overall and item-level fit. The social determinants gender, age, education level, employment status, population density, economic situation, and social status level were included as person factors when testing for differential item functioning (DIF).

**Results:**

A total of 1,064 participants were included in the analysis. Both the Navigational Health Literacy scale (NHL) (*n* = 1,063) and the Digital Health Literacy scale (DHI) (*n* = 926) met the assumption of unidimensionality. Reliability was sufficient for group-level comparisons, although the DHI scale could have been better targeted at respondents’ health literacy levels. Evidence for local independence was partially supported. Data-model fit statistics indicated an acceptable fit for both scales at the overall level (chi-square test) and the item level (infit statistics). However, one item on the NHL scale discriminated poorly and displayed disordered thresholds.

**Conclusion:**

These findings contribute to the ongoing refinement and validation of the NHL and DHI scales within the LTC population and highlight the need for further improvement to strengthen the conceptual understanding of navigating the health care environment and assessing, understanding, applying and using digital health information.

## 1 Introduction

Understanding the global disparities in hospital admission for long-term conditions (LTCs) reveals a pressing public health concern (1). In Europe, a staggering 80% of primary health care users over the age of 45 suffer from LTCs, with nearly half grabbling with comorbidities (2). Amidst these challenges, health literacy (HL) merges as a critical factor, influencing individual’s proficiency to effectively navigate the healthcare landscape and access trustworthy digital health information (3–6). Various HL measurement scales are being validated and applied in different populations. To advance the ongoing initiative, it is essential to validate HL assessment scales across diverse target populations in various contexts (7). This paper aims to contribute to this important discourse by validating HL measures in individuals with LTCs, ultimately fostering better health outcomes for individuals with LTCs. Research have indicated that stronger HL correlates with improved self-management of LTCs (8).

Personal HL is being recognized as one of the most important health determinants (WHO) (9) and refers to the proficiency to access, understand, appraise, and apply health information to make informed decisions about one’s own health and that of others (9). These skills relate to behaviors and lifestyle choices, preventive activities, self-management of diseases, as well as the use of health information and welfare services (10, 11). The public’s options for informed health decisions depend on a sufficient overview of what health care services to use, and will depend on the interaction between individual HL and the nature of the complexity and demands from the health services (12). Navigational HL encompasses the ability to manage information in a way that facilitates optimal navigation in the health care services, and to identify the right treatment or support at the right time (12). Digital HL refers to the ability to seek, find, understand, and appraise health information from digital sources, and to apply this knowledge to solve health challenges (13, 14). Moreover, digital HL involves the skills necessary to utilize digital services and welfare technology (15, 16). Optimizing this capacity to efficiently navigate the health services and utilize digital health services may reduce individual consequences of illness and avoid overburdening the health system (3, 17, 18). LTCs often require a high degree of self-management and patient engagement (19). Individuals living with these conditions have therefore emerged as a key demographic in health promotion and healthcare service planning (17, 20–23), particularly in efforts to achieve sustainable goals related to health and wellbeing for all (22, 24). Furthermore, HL is increasingly understood as a dynamic phenomenon that is shaped by the complexity of individual’s life situations and influenced by a range of contextual factors, including social, cultural, and environmental conditions (10, 25). Thus, individual HL is acknowledged as a relational concept which encompasses both systemic and individual contributors (26). As an asset which can be built through action at an individual, service and societal level (27), a public health approach to HL assessment claims the relevance of collecting data on both comprehensive and specific HL measures as well as sociodemographic variables that comprise the broad complexity that influence daily life decisions on health (28). Taking the considerable and increasing amount of the LTC population into account, increasing HL in LTC populations may make significant contributions towards more health literate populations (26). Validating the scales applied in population surveys is vital in a global context. How the scales operate when applied at different target groups and how each item is perceived by respondents in the target groups is therefore of relevance. Trends in the digitalization of healthcare services and the growing availability of digital health information highlight the importance of incorporating both navigational and digital HL as key assessment outcomes of HL mapping.

A central attention in the field of HL research has been to identify population and patient groups of low HL or high risk of low HL. This earlier strand of HL research found that individuals with LTCs face greater HL demands but often possess lower HL skills to effectively manage their health compared to the general population (29, 30). Associations between lower HL and lower health knowledge, higher decisional uncertainty and low engagement in patients are concerns voiced in several studies (8, 31). This focus on low HL has primarily been addressed for low-to middle income countries (21). As stressed in HL research, public health assessment of the population that is handling health and illness in everyday life is a key priority, also recommending including social determinants and the broader societal context of HL in assessment studies.

Despite research emphasizing that HL is vital key for health promotion and the treatment of LTCs (4, 8, 10, 22, 32, 33) and essential for self-management skills (17, 21), there is a lack of research on HL within LTC populations (11, 17, 18). Nevertheless, the mapping of HL has utilized various specialized HL scales for targeted assessments, such as Navigational Health Literacy (HLS19-NHL_Norwegian) (NHL) and Digital Health Literacy in the domain of Digital Health Information (HSL19-DHI_Norwegian) (DHI), alongside the comprehensive International Health Literacy Population Survey Questionnaire (HLS19). This ongoing research supports evidence-informed health promotion strategies. The primary goal is to obtain valid and reliable HL data to assess and compare populations across regions and countries, as well as to enable longitudinal monitoring. Assessing HL in populations with LTCs is essential to address demographic and technological changes and to guide health care policy development (10, 34–38). However, there remains a significant need for valid and reliable measurement tools specifically tailored to assess NHL and DHL across diverse LTC populations, as these are key to the effective management of such conditions. While NHL and DHI measurement scales have been validated in general populations (39), there is a notable research gap concerning their applicability and validity in populations with LTCs or non-communicable disease (NCDs). Current public HL assessments primarily focus on individual patients in clinical settings and do not sufficiently capture population-level HL mapping within specific contexts or subgroups (27). Crucially, it remains unclear whether population surveys yield valid measurements for individuals with LCTs. Existing HL measurement studies in LTC populations typically focus narrowly on particular diagnosis or clinical subgroups (40), rather than reflecting the full diversity of the broader LTC population (8, 26). This limitation reveals a significant gap in knowledge and underscores the importance of adopting a population-based perspective that includes a more heterogeneous sample of self-reported LTCs (27, 40, 41).

Moreover, although NHL and DHI measurement scales have been validated in general populations (7, 16) and specific target groups (42), their applicability has not yet been examined among individuals with LTCs. Traditional validation methods, particularly confirmatory factor analysis (CFA) (40), continue to dominate the HL field. However, recent studies highlight the advantages of applying Rasch modelling in achieving specific objectivity (16, 39, 43–46).

CFA models for categorical data allow varying item loadings, and item response theory (IRT) models allow items to differ in discrimination, whereas Rasch models constrain discrimination parameters to be equal across items. This constraint results in independence between person parameters (what is being measured) and item parameters (the measurement tool), a property known as specific objectivity. IRT models, including Rasch models, place items and persons on the same measurement scales and compare the distribution of person proficiency to the distribution of item thresholds.

In heterogeneous samples of individuals with LTC drawn from the general population, where both proficiency and interpretations of item content may vary, it is important to examine whether HL scales measure invariantly across the general population and a heterogeneous subgroup of individuals with LTCs. In line with these recommendations, this study applies the Rasch model to enhance the measurement of HL in heterogenous LTC populations.

## 2 Methods

### 2.1 Aim

The aim of this study was to explore the psychometric properties of the measurement scales used to assess of Navigational Health Literacy (HLS_19_-NHL_Norwegian) (NHL) and Digital Health Literacy within the dimension of Digital Health Information (HLS_19_-DHI_Norwegian) (DHI) in a population sample of individuals with long-term conditions (LTCs). The data collected was tested against the Rasch partial credit parametrization (PCM) of the unidimensional Rasch model for polytomous responses (47). More specifically, we examined whether the data generated by the scales were consistent with the following evaluative statements, which we formulated as hypotheses:

H1: The measurement scales collect data that meets the unidimensional Rasch model requirements of unidimensionality and acceptable reliability and display proper targeting with no violation of local independence.

H2: Each item displays sufficient data-model fit, with ordered response categories.

### 2.2 Design

This study is a secondary analysis of survey data collected in Norway as part of the international large-scale European Health Literacy Population Survey 2019–2021 (HLS_19_) (36, 39, 48). Administered by the WHO Action Network on Measuring Population and Organizational Health Literacy (M-POHL) of Europe, HLS_19_ mapped HL across 17 countries (39, 49, 50). In Norway, the data were collected in 2020 by a national survey agency using computer assisted telephone interviews (CATI). The response rate calculated as the number of individuals contacted was at 20%, and further details regarding sampling and representativeness are reported elsewhere (36, 48). The analysis adhere to the STROBE guidelines for cross-sectional studies (51) as well as the Rasch reporting guideline for rehabilitation research (52).

### 2.3 Participants

The current study included 1,064 respondents ≥18 years who reported having one or more LTCs (Table 1). This target sub-population was selected from the general population, constituting a heterogeneous sample of diverse LTCs, including musculoskeletal conditions and hypertension, cardiovascular diseases, diabetes mellitus, mental illness, lung disease, and rare diseases. A total of 3,000 participated in the national survey; however, 94 were excluded from our sample of adults (89 reported being under 18, and five had missing data). Additionally, respondents with missing values regarding LTCs were excluded (*n* = 24). Among the individuals with self-reported LTCs (*n* = 1,064), *n* = 1,063 responded to at least one NHL-item and *n* = 926 responded to at least one DHI item. The latter sample size was lower as those who responded “no” on the routing item about whether they had ever used the internet to search for health information (36), were excluded from the DHI analyses.

**Table 1:** Sample characteristics of sub-sample with self-reported LTCs compared to the sub-sample reporting no LTCs.

Sociodemographic variables	LTCs	Mis.	No LTCs	Mis.
*n*	1,064		1818	
Gender				
Female	56	-	46	-
Male	44	-	54	-
Age cat ≤ 65	74		83	-
> 65	26		17	
Education level		2		-
ISCED 0─5	50		46	
ISCED 6─8	48		52	
Employment status				
Employed	61	-	80	-
Unemployed	39		20	
Population density (urbanity)		-		1
≥50.000	40		45	
<50.000	60		54	
Economic deprivation		1		1
No	49		56	
Yes	50		43	
Social status level		4		3
Low (1─4)	11		7	
High (5─10)	86		90	

Sub-sample with self-reported LTCs (*n* = 1,064) compared to the sub-sample reporting no LTCs (*n* = 1818) in a representative national sample (Norway) of the cross-sectional HLS19-data. All numbers reported in %, except *n*. Education level: ISCED 5 ≤ short-cycle tertiary education/ISCED 6 ≥ bachelor’s or equivalent; Employment status: ‘unemployed’ entails unemployed, retired and unable to work; Economic deprivation: ‘Yes’ entails very difficult to pay bills at the end of the month, Social status operationalized as ten-level variable in HLS_19_, split between level four and five to indicate low or high perceived social status.

### 2.4 Measurement scales

The culturally adapted versions of the NHL and DHI-measurement scales, based on work by M-POHL and further developed by the Norwegian HLS_19_ national study team, consists of 12 and eight items, respectively. A four-point self-report rating scale graded 0─3 (very easy, easy, difficult and very difficult) was used for all items (36, 48). NHL is conceptually balanced between organizational- and system level items. DHI reflects the domain of health information on the Digital Health Literacy scale (36, 48). The item statements and domains are presented in Table 2.

**Table 2:** Item labels, statements and domains in the NHL and DHI measurement scales.

Item label	Statement (“on a scale from very easy to very difficult: how easy would you say it is to…?”)	Domain
NHL1	Understand information about how the health care system is structured and is functioning	System
NHL2	Determine what type of health care you need when you have a health problem	System
NHL3	Determine whether health insurance covers your need for a particular health service	System
NHL4	Understand information about ongoing health care reforms that may impact your health care services	System
NHL5	Find out what rights you have as a patient or user of health services	System
NHL6	Determine what health services to choose if you need one	Organizational
NHL7	Find information about the quality of a particular health service	Organizational
NHL8	Determine whether a particular health service meets your need for health care	Organizational
NHL9	Know how to book an appointment at the primary health service	Organizational
NHL10	Find out how patient and user organizations or similar can help you navigate the health care system	Organizational
NHL11	Find the right contact person for your needs at a health institution	Organizational
NHL13	Find out if a health service requires a deductible	System
DHI1	Use the proper words or search query to find the information you are looking for	Digital
DHI2	Find the exact information you are searching for	Digital
DHI3	Understand the information	Digital
DHI4	Judge whether the information is reliable	Digital
DHI5	Judge whether the information is offered with commercial interests	Digital
DHI6	Visit different websites to check whether they provide similar information about a topic	Digital
DHI7	Judge whether the information is applicable to you	Digital
DHI8	Use the information to help solve a health problem	Digital

NHL12 was removed and replaced by NHL13 as part of the national validation.

### 2.5 Rasch model estimation

We explored the proposed hypotheses by applying the Rasch partial credit parametrization (PCM) (47) of the unidimensional Rasch model to test the psychometric properties of navigational and digital HL scales in the LTC population. The analyses were performed using Rumm2030 Plus (53), employing pairwise maximum likelihood estimation for item parameters and subsequently Warm’s weighted likelihood estimation for person parameters (54). Additionally, to further evaluate individual item fit and overall data-model fit, we utilized the Mirt R package using marginal maximum likelihood estimation (55–57).

### 2.6 Handling missing data

Individuals with missing values for all scale items were not included in the analysis. The number of participants with missing values differed between the NHL and DHI scales. The scales were analyzed separately, and respondents who responded “no” to a routing question about using internet to search for health information, were not asked the DHI items.

### 2.7 Rasch model application

Using Akaike Information Criterion (AIC) (58), we identified and selected the most appropriate Rasch model for polytomous items. The PCM described the data better than the Rating Scale Model (RSM), as the AIC decreased by 65.4 for the NHL scale and by 48.9 for the DHI scale when the PCM was applied. Therefore, the PCM was retained for all subsequent Rasch model applications.

#### 2.7.1 Unidimensionality

To test the assumption of unidimensionality (59), dependent *t*-tests were used to estimate the portion of respondents with statistically significantly different proficiency estimates based on identified subscales (target value is < 5%). For the NHL scale, we used the system level and the organizational level item subsets as theoretically defined subscales (Table 2), while we used principal component analysis (PCA) of Rasch-model residuals (59) to empirically identify possible subscales for the DHI scale. Dependent *t*-tests were also used to confirm the two-dimensional structure of the composite scale combining NHL items and DHI items.

#### 2.7.2 Reliability

Scale reliability was estimated by the Person Separation Index (PSI) (60) and marginal reliability index. Acceptable reliability coefficients should preferably be minimum 0.65 at the group level (61).

#### 2.7.3 Targeting

Scale targeting was explored by comparing the distribution of person proficiencies (which represents the respondents scale score) to the distribution of item thresholds (indicating the ‘difficulty’ of endorsing the various response categories), where the mean of the latter was constrained to 0. To which extend person ability mean exceeds below or above mean indicate whether the respondents find the scale more easy or difficult than intended, and the mean position indicates how the distribution of person proficiencies match the distribution of item thresholds. As the item mean is fixed at 0, positive mean for person estimates indicate that the items in the scale are easy on average. The standard deviation of logits for person estimates indicates the spread of person measures on each scale (52, 62).

#### 2.7.4 Local independence

Measurement scales may exhibit response-level and trait-level violations, including violations of the assumption of local independence (LID) (63). The assumption of invariance was tested using differential item functioning (DIF). When items display DIF, they function differently across groups, indicating a lack of invariance. DIF can also be interpreted as a trait-level violation, as the test identifies factors associated with the latent trait. An item displays differential item functioning (DIF) if respondents with the same proficiency from different groups differ in their response patterns (e.g., the two group levels males and females for the person factor gender).

The DIF analyses tested whether either the slope of the item characteristic curve (indicating non-uniform DIF) or the locations of the item thresholds (indicating uniform DIF) varied across the level of the relevant person factor (64). Using analysis of variance of standardized Rasch model residuals (65) with Bonferroni adjusted 5%-level (66), DIF was tested for gender, age, education level, employment status, population density, economic situation and social status (Table 1). While the target value for chi-square probability equals 0.05 divided by number of items (e.g., 0.05/12 for NHL), the chi-square target value applied for the DIF analysis was divided by two categories (non-uniform and uniform DIF) (e.g., 0.05/24 for NHL). Age was dichotomized in line with applied age categorization in health promotion (67) and the definition of older adults (65 or older/below 65). Education level was split between ISCED (International Standard Classification of Education) levels 5 and 6 (6 = bachelor’s or equivalent) (68). Employment status was recoded to either employed or unemployed, with the unemployed category including those who are unemployed, retired or who reported that they were unable to work due to long-standing illness. Population density was recoded from four categories to a binary classification of above or below 50.000 inhabitants. Economic situation, also known as economically deprivation, was recoded from four categories (very easy, easy, difficult or very difficult *to pay bills at the end of the month*) into a dichotomous variable classified as ‘Pay bills’ (very easy versus easy/difficult/very difficult). Social status, initially defined as an eleven-level variable in HLS_19_ (36) was split between level four and five to indicate low or high perceived social status. Person factors were selected to address the recognized need to include sociodemographic variables in health literacy (HL) assessment, given concerns about social gradients in HL development (69). Dichotomization was performed using optimal cut-off points.

#### 2.7.5 Data-model fit

Data-model fit was tested by Bonferroni-adjusted chi square fit statistics at the overall level and at item level infit values, and graphical inspection of item characteristic curves. The full sample sizes were used for all analyses (*n* = 1,063 for NHL and *n* = 926 for DHI), but adjusted sample sizes corresponding to 10─30 persons per threshold (62, 70, 71) were applied for DIF analyses and analyses of data-model fit at item level and overall scale level as chi square fit statistics are sample-dependent (72). The probability of observing a given chi-square value or a larger value under the assumption of good model fit is indicated by a non-significant chi-square test. The expected value of Infit mean square (MNSQ) is 1 (55), under the hypothesis that the PCM explains all the variance in the item responses (73). Infit values between 0.7 (strong discrimination) and 1.3 (weak discrimination) are considered sufficient (10, 39), and indicate that there is 30% more or less variation in the data than explained by the PCM, respectively (74). Ordered uncentralized item thresholds were interpreted as evidence for ordered response categories (47). The assumption of local independence was was tested by estimating correlations between Rasch-model residuals (Yen’s Q3) for each pair of items (65, 75) also known as ‘response dependence’ and ‘response violation of local independence’ (63).

To further evaluate model adequacy, goodness-of-fit indices (GOFIs) are reported for the PCM, including the standardized root mean squared residual (SRMR) with target value < 0.08 (76), the root mean squared error of approximation (RMSEA) with target value < 0.05 (77), and the Comparative fit index (CFI) and the Tucker-Lewis index (TLI) with target value > 0.95 (78, 79). The target values are valid for SEM-based CFA with continuous indicators and should be interpreted with cautious when applied to item response theory (IRT) models. In the current analyses of polytomous data, we applied M2* specified by C2 which is conceptually comparable to chi-square fit index for CFA (56, 57). These estimates are reported as a supplementary analysis for descriptive and comparative purposes.

## 3 Results

The analysis provides partial support for H1, which concerns the evaluative criteria of unidimensionality and reliability, and proper targeting and local independence when applied in the population of individuals with long-term conditions (LTCs). The first part of H1 was largely supported across both scales, with evidence of sufficient unidimensionality and reliability. Local independence (LID) was partly supported, as no substantial response dependency was observed, but compromised by items displaying differential item functioning (DIF). H1 was further weakened in the Digital health literacy scale (DHI), where poor person–item targeting was observed. H2, addressing data-model fit and threshold ordering, indicated item-level issues in the Navigational health literacy scale (NHL).

### 3.1 Unidimensionality

For individuals with LTCs, both scales sufficiently met the requirement of unidimensionality, with fewer than 5% of dependent *t*-tests being statistically significant.

### 3.2 Reliability

We observed sufficient reliability for both scales, with the person separation index (PSI) and Cronbach’s alpha exceeding 0.85.

### 3.3 Targeting

**Figure 1:**
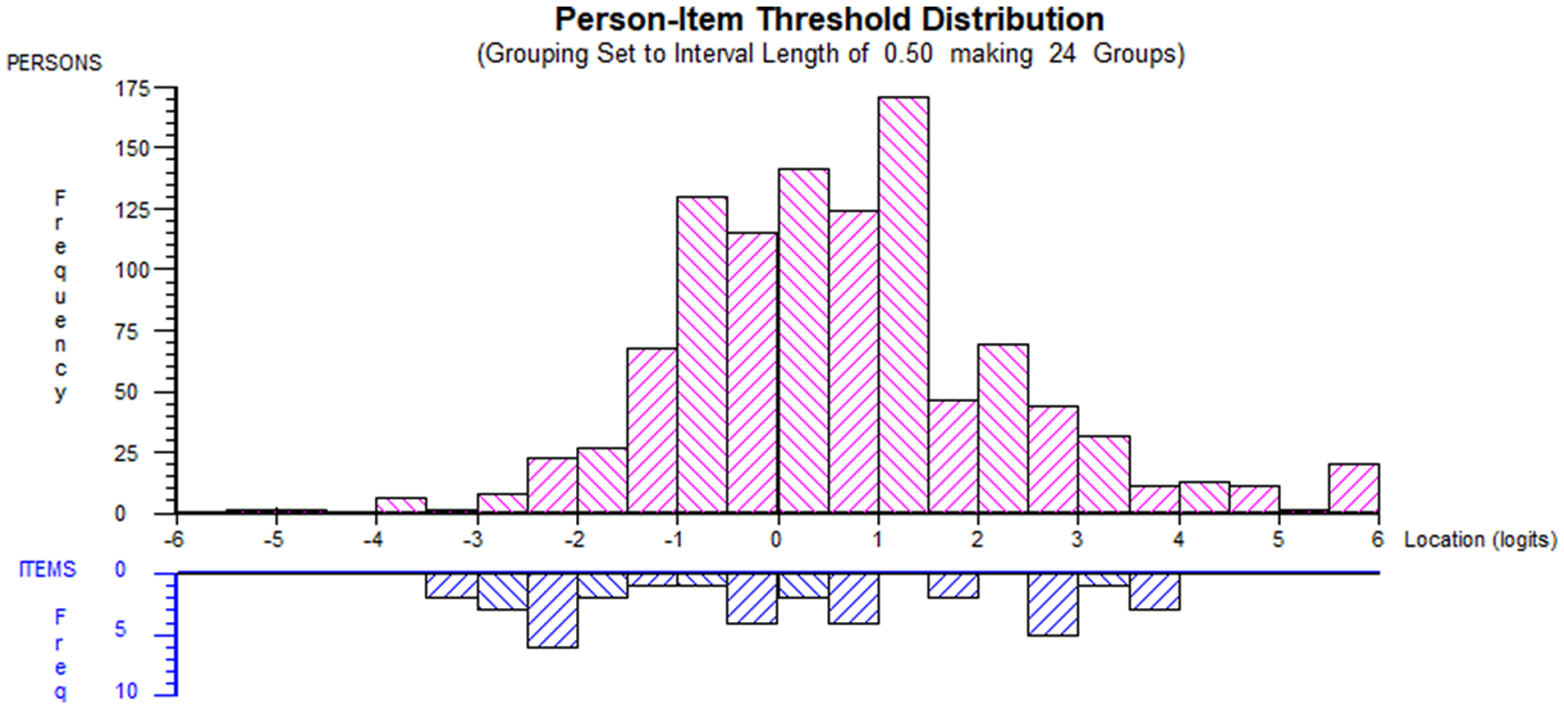
Threshold-item distribution for HLS_19_-NHL_Norwegian distribution of person estimates (red histogram, *n* = 1,063, *m* = 0.65, *sd* = 1.69) and item thresholds (blue histogram, *n* = 36, *m* = 0.00, *sd* = 0.74) with information function (green line) for HLS_19_-NHL_Norwegian (NHL) when applied in the sub-population of people with self-reported long-term conditions (LTCs) (generated using Rumm 2030).

**Figure 2:**
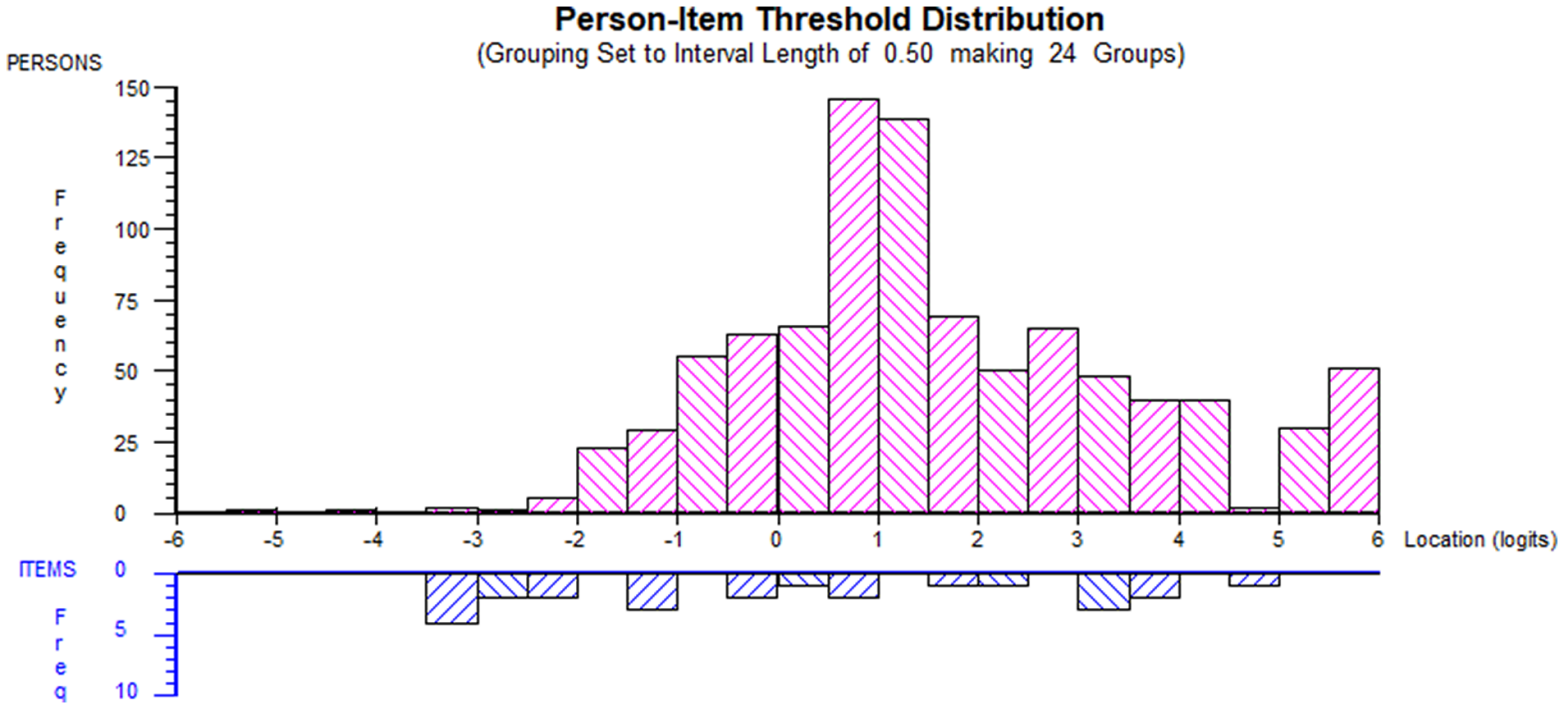
Threshold-item distribution for HLS_19_-DHI_Norwegian Threshold-item distribution for HLS_19_-DHI_NorwegianDistribution of person estimates (red histogram, *n* = 926, *m* = 1.63, *sd* = 1.97) and item thresholds (blue histogram, *n* = 24, *m* = 0.00, *sd* = 0.60) with information function (green line) for HLS_19_-DHI_Norwegian (DHI) when applied in the sub-population of people with self-reported long-term conditions (LTCs) (generated using Rumm 2030).

The NHL scale was better targeted to the LTC population than the DHI scale (Table 3). Graphical representations of the threshold-person distribution are provided in Figure 1, 2, confirming the slightly skewed distribution of the DHI scale. Both item sets are presented in Table 4 in ascending order of ‘overall item location’, indicating the ordering of HL-related tasks from the ‘easiest’ to the most ‘difficult’.

**Table 3:** Overall scale level results for HLS_19_-NHL and HLS_19_-DHI Norwegian versions in the LTC population.

Statistics	HLS_19_-NHL *n* = 1,063	HLS_19_-DHI *n* = 926
Unidimensionality % sign. Tests (CI)	6.42 (0.05)	5.56 (0.04)
Chi-square *χ*^2^(*df, n*) *p*	284.36 (108, 1063) < 0.00	129.99 (72, 926) < 0.00
Chi-square amend *sample χ*^2^(*df, n*) *p*	133.41 (108, 487) < 0.05	92.76 (72, 623) < 0.05
Mean person location *m* (*sd*)	0.65 (1.69)	1.63/1.97
Min and max item threshold	−3.31; 4.51	−3.50;3.83
Person separation index (PSI) α ^MIRT^	0.89 0.92	0.86 0.89
SRMSR ^MIRT^	0.09	0.07
RMSEA ^MIRT^	0.10	0.10
M2 RMSEA *p* value [x^2^(*df*)*p*] ^MIRT^	717.10(65)0	273.17(27)0
CFI ^MIRT^	0.95	0.96
TLI ^MIRT^	0.94	0.96

m/sd and thresholds in logits; SRMSR Standardized Root Mean Square Residuals RMSEA; Root Mean Square Error of Approximation; CFI Comparative Fit Index; TLI Tucker-Lewis Index; M2 C2; n sample size for DHI is reduced by inclusion criteria (“have you ever searched for health information on the internet”); extreme scorers are included in Rumm estimates. Dimensionality between NHL and DHI [% sign. Tests (CI) = 23.19 (0.02)].

**Table 4:** Item statements (ascending location order, from the easiest to the most difficult) for LTC population.

Item label	Statement (“on a scale from very easy to very difficult: how easy would you say it is to…?”)
NHL 9	Know how to book an appointment at the primary health service
NHL 2	Determine what type of health care you need when you have a health problem
NHL 13	Find out if a health service requires a deductible
NHL 6	Determine what health services to choose if you need one
NHL 1	Understand information about how the health care system is structured and is functioning
NHL 11	Find the right contact person for your needs at a health institution
NHL 10	Find out how patient and user organizations or similar can help you navigate the health care system
NHL 8	Determine whether a particular health service meets your need for health care
NHL 3	Determine whether health insurance covers your need for a particular health service
NHL 5	Find out what rights you have as a patient or user of health services
NHL 7	Find information about the quality of a particular health service
NHL 4	Understand information about ongoing health care reforms that may impact your health care services
DHI 1	Use the proper words or search query to find the information you are looking for
DHI 6	Visit different websites to check whether they provide similar information about a topic
DHI 3	Understand the information
DHI 2	Find the exact information you are searching for
DHI 7	Judge whether the information is applicable to you
DHI 5	Judge whether the information is offered with commercial interests
DHI 4	Judge whether the information is reliable
DHI 8	Use the information to help solve a health problem

### 3.4 Local independence and invariance

Local response independence was supported, as Q3 values were below 0.2 in both scales. However, the assumption of local independence (LID) was weakened by evidence of trait-violation. Using reduced sample size (*n* = 487 for NHL and *n* = 623 for DHI), item NHL 1 displayed DIF by education level in favor of respondents with higher education, and item DHI 6 displayed DIF by age group in favor of the younger respondents. When applying full sample (*n* = 1,063 for NHL and *n* = 926 for DHI) NHL 1 and NHL 4 displayed DIF by employment status, NHL 7 by education level, NHL 9 by economic deprivation in the NHL scale, and DHI 4 by employment status and DHI 7 on age group in the DHI scale. A nuanced understanding of differential item functioning (DIF) requires graphical inspections, which are further elaborated in the discussion section.

### 3.5 Data-model fit

**Figure 3:**
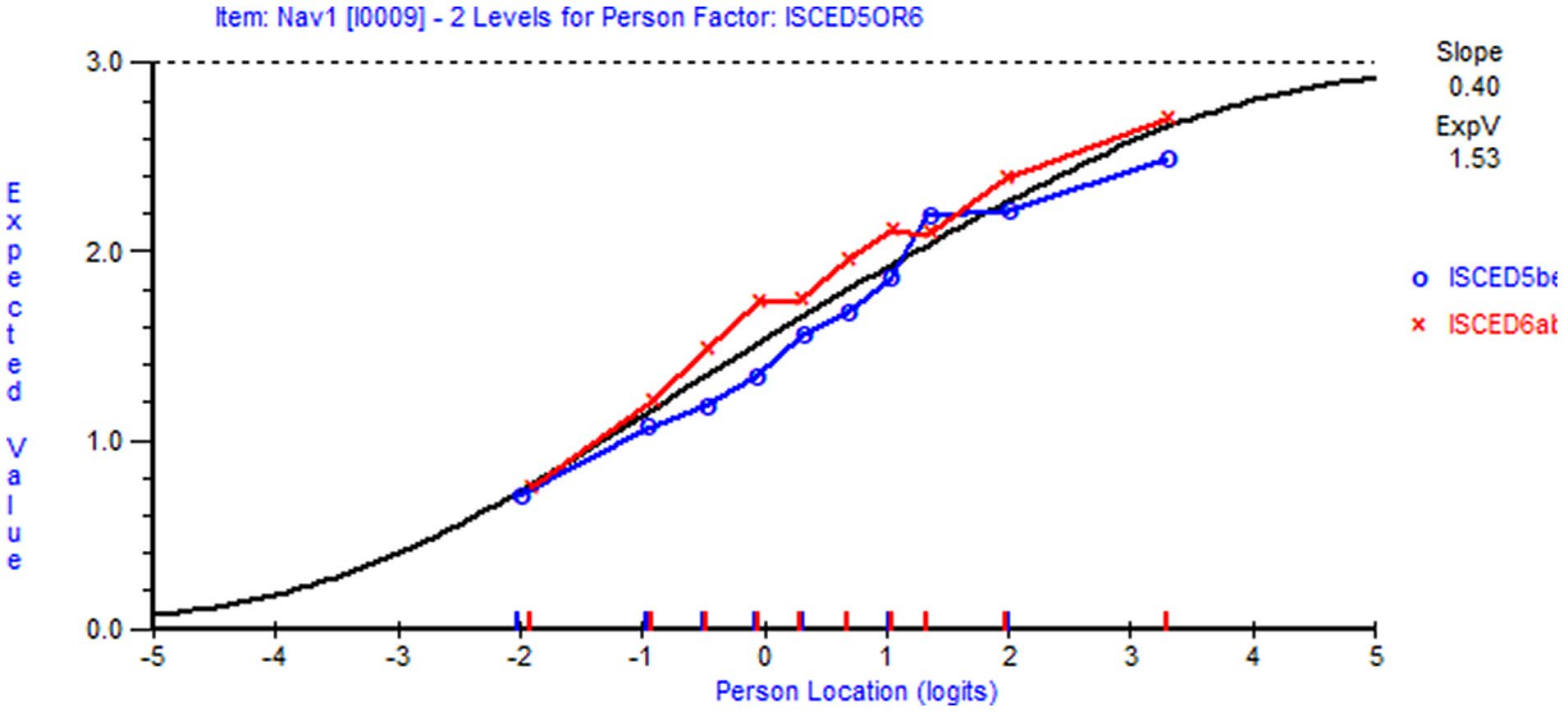
DIF by education in item NHL 1. DIF by education (ISCED 5/6) for item NHL 1 To understand information about how the health care *system is structured and is functioning (loc=-.00; Chi Sq (p)=2.08 (.99); Infit=.91; missing=4.5 %)* (generated using Rumm 2030).

Overall data-model fit was acceptable for both scales (Table 3). For the 12-item NHL scale (*m* = 36 thresholds), the chi-square test was statistically non-significant (*p* > ≈ 0.05) when the sample size was reduced from 1,063 to 487 respondents (i.e., 14 respondents or >10 respondents per threshold). Similarly, for the 8-item DHI scale (*m* = 24 thresholds), the chi-square test was non-significant when the sample size was reduced from 926 to 623 respondents (i.e., 27 respondents or >20 respondents per threshold). At the item level, chi-square test statistics (with Bonferroni-adjustment) revealed that one NHL item (NHL 9) and one DHI item (DHI 5) discriminated somewhat poorly and that two NHL-items over-discriminated relative to the Rasch model expectations (NHL 5 and NHL 8). When the sample size was reduced to the recommended range of 10–30 persons per threshold (80). All items but NHL 9 displayed sufficient data-model fit. Despite these issues, graphical inspections of item characteristic curves (ICCs) showed that all items functioned sufficiently. At the item level, all infit values fell within the acceptable range for survey data (39) (0.72–1.25 for NHL items and 0.80–1.02 for DHI items) (Table 5). Mean person location for NHL was 0.65 (*sd* 1.69) and 1.63 (*sd* 1.97) for DHI (Table 3). For the DHI scale, all items demonstrated ordered response categories. However, NHL 9 displayed unordered thresholds (Figure 3). Except for a somewhat large RMSEA-value for the DHI scale when estimating the PCM by using the Mirt R-package, the supplementary CFA-based fit indices SRMR, RMSEA, CFI and TLI strengthened the idea of sufficient overall data-model fit (Table 3).

**Table 5:** Item location estimates, item fit, LD and DIF for HLS_19_-NHL and HLS_19_-DHI in the LTC population.

Item label	Item loc	Chi Sq	(p)	Infit ^MIRT^	LD	DIF ^*^	% Miss
HLS_19_-NHL							
NHL 9	−1.62	47.74	**(0.00)**	1.25	-		0.7
NHL 2	−0.89	5.54	(0.79)	0.85	-		2.1
NHL 13	−0.50	9.90	(0.04)	1.05	-		5.6
NHL 6	−0.29	4.13	(0.90)	0.85	-		6.0
NHL 1	−0.00	2.08	(0.99)	0.91	-	Education	4.5
NHL 11	0.05	3.62	(0.94)	0.89	-		8.3
NHL 10	0.06	2.23	(0.99)	1.00	-		16.0
NHL 8	0.39	19.68	(0.02)	0.72	-		6.6
NHL 3	0.49	10.99	(0.28)	1.07	-		17.0
NHL 5	0.50	11.38	(0.25)	0.77	-		4.6
NHL 7	0.86	12.89	(0.17)	0.83	-		7.0
NHL 4	0.96	3.23	(0.96)	0.86	-		9.0
HLS_19_-DHI							
DHI 1	−0.83	9.49	(0.39)	0.93	-		1.2
DHI 6	−0.70	10.40	(0.32)	0.89	-	Age	3.5
DHI 3	−0.40	10.60	(0.31)	0.81	-		0.6
DHI 2	−0.00	9.10	(0.43)	0.88	-		0.3
DHI 7	0.19	13.80	(0.13)	0.80	-		2.2
DHI 5	0.29	16.70	(0.05)	1.02	-		2.5
DHI 4	0.66	8.30	(0.50)	0.82	-		1.7
DHI 8	0.79	14.30	(0.11)	0.89	-		5.2

Item labels in location order from easiest to most difficult tasks. Item loc mean of uncentralized thresholds in logits.

Chi square (probabilities) < Bonferroni adjusted 0.05% level (<0.006250 for DHI items; < 0.0042 for NHL items) statistically significant deviations in bold; Infit statistics PCM by full sample in Mirt R-package; LD, local dependence; DIF, differential item functioning; Educ, education level with ISCED classification; missing population survey data. *All reported DIF is unform and statistically significant when Bonferroni adjustment at the 0.05 significance level was applied (DIF NHL< 0.002083; DIF DHI <0.003125).

## 4 Discussion

### 4.1 Main findings

This study evaluated the Norwegian versions of the HLS_19_-NHL (Navigational Health Literacy; NHL) and HLS_19_-DHI (Digital Health Literacy; DHI) scales in a sample of 1,064 individuals with long-term conditions (LTCs). Rasch model applications revealed measurement issues that warrant further exploration. Applying the Rasch Partial Credit Model (PCM) for polytomous data, both measurement scales sufficiently met the requirement of unidimensionality and displayed acceptable overall data-model fit for reduced sample size of 10 to 30 respondents per threshold. Nonetheless, both scales showed evidence of measurement non-invariance, as indicated by differential item functioning (DIF) by person factors. Because this was an evaluation of instruments applied in a cross-country large-scale survey in ongoing initiative to map population health literacy (HL), we did not collapse categories in NHL 9 or remove items from the scale to avoid DIF. Ordered response categories were observed for the DHI scale, but the DHI scale was less well-targeted as compared to the NHL scale. These results underline the need to further refine the scales, so they more fully meet the requirements for objective measurement. The model was not optimal, however, our intention in applying the Rasch model was not to revise the measurement scales items but to apply an IRT-based approach to identify potential issues. Instances of misfit proved valuable insights to guide further refinement and data collection.

#### 4.1.1 Navigational health literacy (HLS_19_-NHL_Norwegian) (NHL)

Applied to the LTC population, the NHL measurement scale was sufficiently unidimensional. Reliability indices were sufficient, the scale displayed acceptable targeting, and no substantial response dependency was observed. However, non-invariance due to DIF by education indicated a possible violation of local independence. While overall data-model fit was satisfactory, the analysis revealed problems at the item level. One item (NHL 9) requires attention, as it shows weak discrimination and unordered thresholds. However, the overall test situation with infit values and graphical inspections strengthened the idea of sufficient data-model fit at the item level. CFA-based indices were supportive.

#### 4.1.2 Digital health literacy in the domain of digital health information (HSL_19_-DHI_Norwegian) (DHI)

The DHI scale demonstrated sufficient unidimensionality and reliability, but the scale demonstrated somewhat poor targeting. The assumption of no response violation of local independence was met, but one item displayed DIF by age. Overall- and item level fit statistics were sufficient.

### 4.2 Implications for NHL and DHI measurement

Consistent with findings from prior research (36), the scales could have been better targeted, as few item thresholds were located at higher standing on the latent trait (Figures 1, 2). However, the marginal reliability estimates were sufficient. Despite some limitations, both scales proved acceptable reliability, coherent with validation studies in other European countries (16).

**Figure 4:**
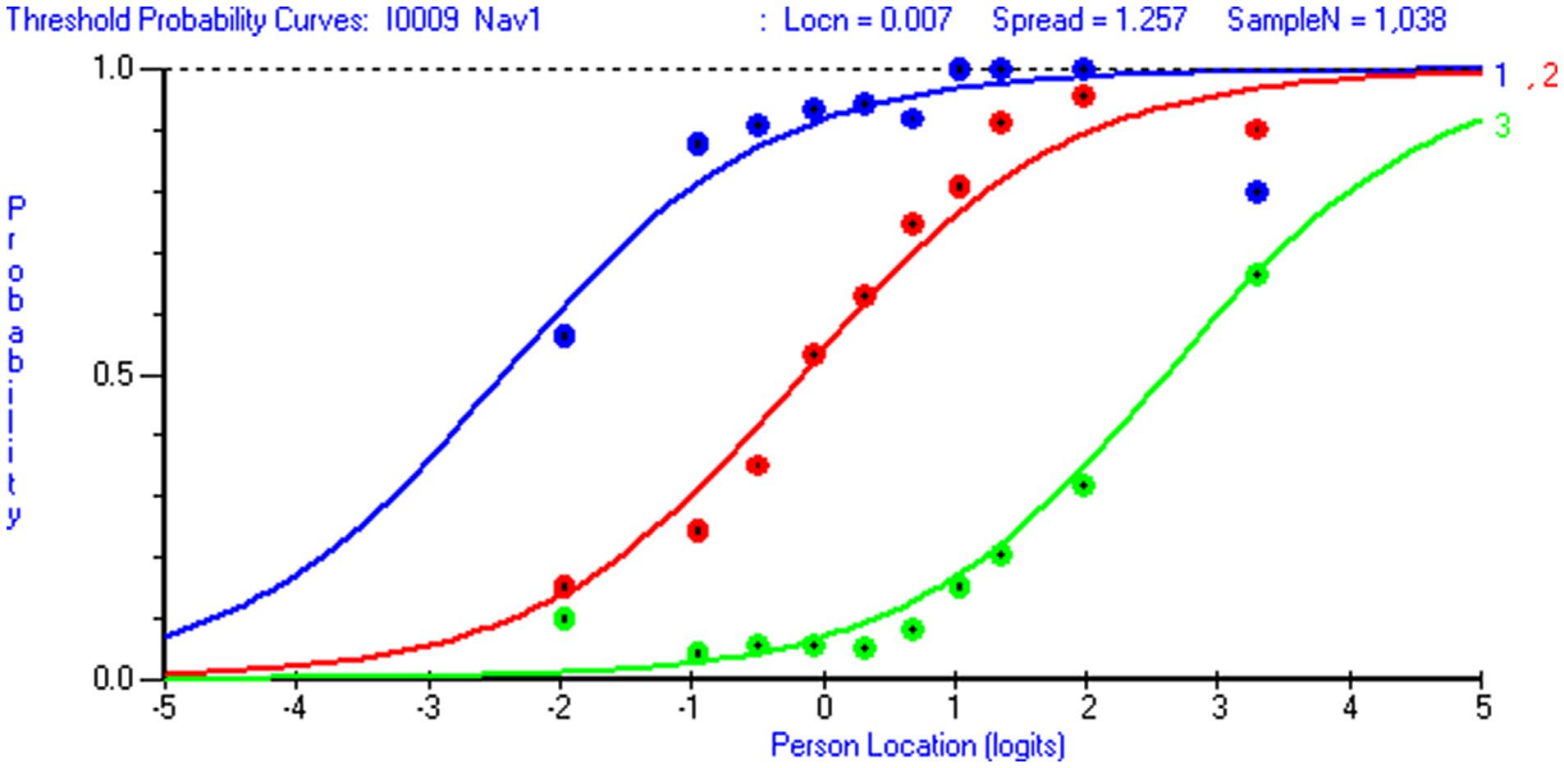
Threshold probability curves in item NHL 1. Threshold probability curves with observed data in item NHL 1 To understand information about *how the health care system is structured and is functioning (loc=-.00; Chi Sq (p)=2.08 (.99)*; Infit=.91; missing=4.5 %) (generated using Rumm 2030).

**Figure 5:**
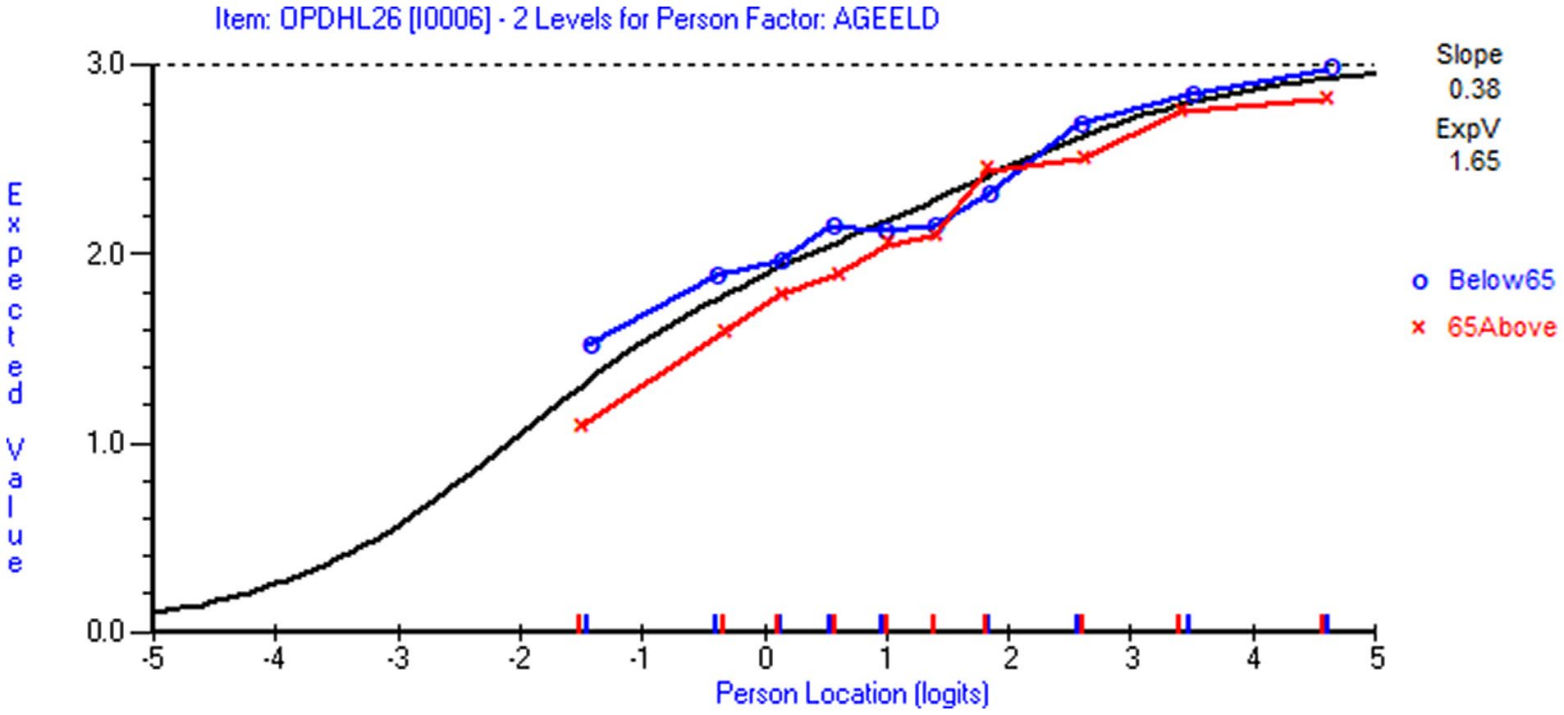
DIF by age for item DHI 6. DIF by age (below 65/65 and above) for item DHI 6 To visit different websites to check whether they *provide similar information about a topic (loc=-.70; Chi Sq (p)=10.40 (.32); Infit=.89; missing=3.5 %)* (generated using Rumm 2030).

**Figure 6:**
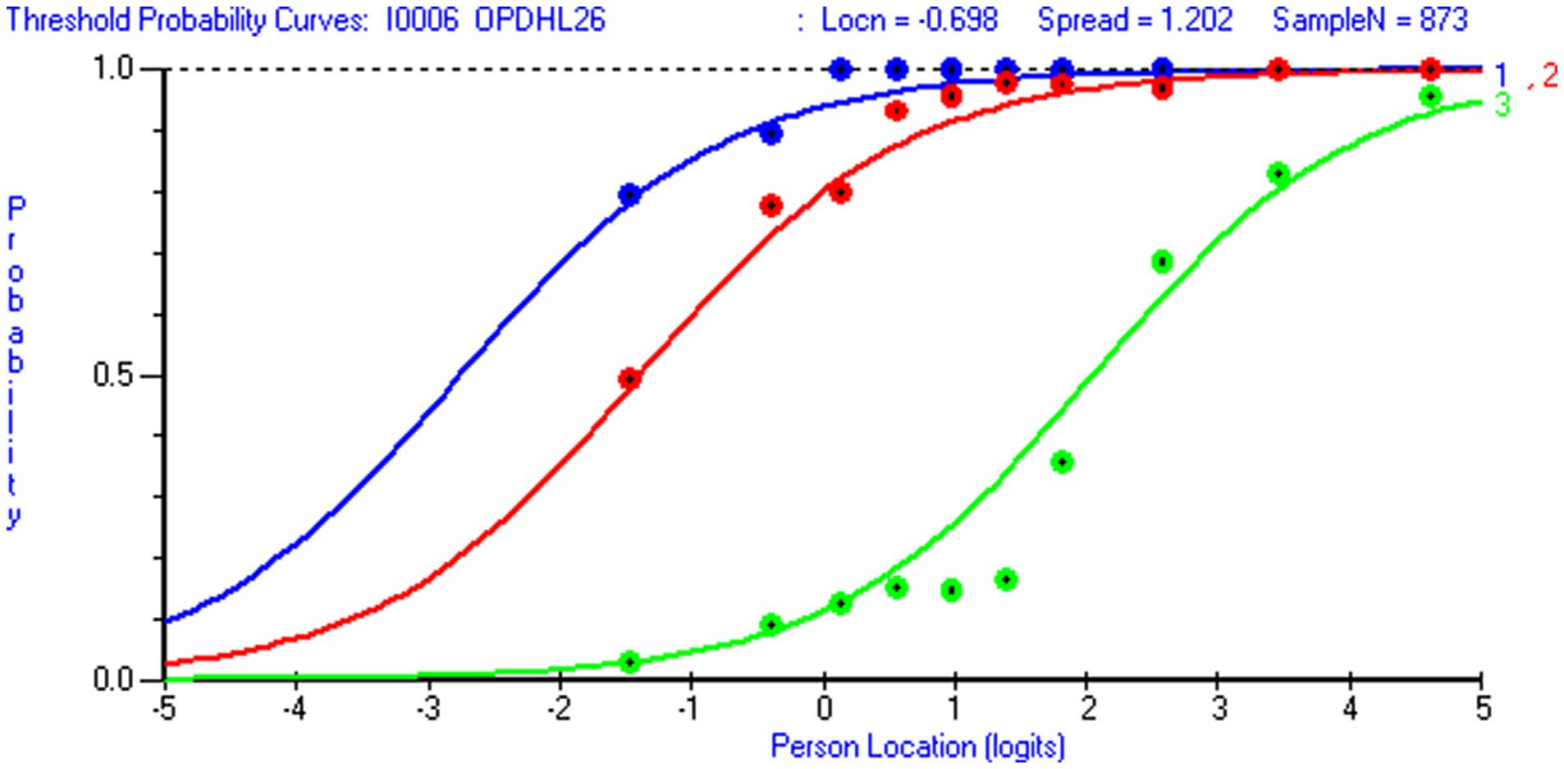
Threshold probability curves in item DHI 6. Threshold probability curves with observed data for item DHI 6 To visit different *websites to check whether they provide similar information about a topic (loc=-.70; Chi Sq (p)=10.40 (.32); Infit=.89; missing=3.5 %)* (generated using Rumm 2030).

For both scales, we observed trait violations of invariance as some items displayed DIF. Given that HL in general populations (81) is shaped by various factors and casual pathways (82), the distribution of DHI and NHL in the subpopulation of individuals living with LTCs likely reflect contextual factors and person factors, such as access to digital resources, socioeconomic status, educational attainment and employment status. Respondent’s age is a confounder that possibly influences the outcome (DHI and NHL) as well as other person factors. Therefore, it is of particular interest that item NHL 1 (*understanding information about how the health care system is structured and is functioning*) displayed uniform DIF by education level (Figure 4) in favor of respondents with higher education level, and that item DHI 6 (*to visit different websites to check whether they provide similar information about a topic*) displayed uniform DIF by age in favor of the younger respondents (Figure 5). However, detected DIF needs to be interpreted considering the overall test situation. First, items NHL 1 (Figure 4) and DHI 6 (Figure 5) discriminated well (Table 5). Second, unlike health determinants such as gender, education and age do not reflect intact groups but may vary over time. As reported in Table 6, the uncentralized thresholds were increasing in line with this recommendation along the DHI trait. Regarding DIF by age, it is also noteworthy that older population are overrepresented in the LTC sample (Figure 6). This may indicate a selection on the age variable, which is a factor potentially associated with DHI. Conversely on the NHL scale, NHL 9 displayed unordered thresholds (Figure 3). This may consequently be attributable to the wording of the item and the low difficulty level with few respondents in the lower thresholds and thereby limited information (83). Item NHL 9 (*knowing how to book an appointment at the primary health service*) also discriminated somewhat poorly, indicating that the item not shows a positive monotonic relationship with the underlying latent trait (44), suggesting that it may tap into other constructs (84). As displayed in Table 5, item NHL 9 has the lowest item location estimate, and the lowest thresholds are located where few respondents stand on the latent trait. Unordered thresholds may imply that the respondents do not respond consistently with the underlying construct (85). In our view it is neither highly problematic nor unexpected that the two lowest item thresholds are unordered. Still, to meet the fundamental requirements for the scale to operate satisfactorily, this item warrants closer attention and comparisons across groups should be interpreted with caution. Based on our analysis, we recommend further refinement of this item wording and its conceptual clarity in the LTC population, to avoid inconsistent responses that may compromise the validity of the NHL scale.

**Table 6:** Item uncentralized thresholds, proportion and frequency.

Statement	Thr 1	Thr 2	Thr 3	Cat. 1	Cat. 2	Cat. 3	Cat. 4	Frequency
HHL 1	−2.43	−0.16	2.60	0.06	0.31	0.47	0.15	993
NHL 2	−3.49	−1.16	1.99	0.02	0.19	0.56	0.23	1017
NHL 3	−1.72	0.54	2.66	0.11	0.40	0.38	0.12	860
NHL 13	−2.59	−0.71	1.80	0.04	0.22	0.50	0.24	981
NHL 4	−1.51	0.78	3.60	0.14	0.42	0.37	0.07	945
NHL 5	−2.10	0.57	3.00	0.10	0.41	0.39	0.11	993
NHL 7	−2.00	0.74	3.83	0.11	0.44	0.40	0.06	967
NHL 8	−2.64	0.08	3.73	0.06	0.36	0.50	0.08	971
NHL 6	−3.26	−0.24	2.62	0.03	0.32	0.50	0.15	976
**NHL 9**	**−2.31**	**−2.79**	**0.24**	**0.01**	**0.04**	**0.44**	**0.51**	1031
NHL 10	−2.38	−0.16	2.72	0.06	0.32	0.49	0.14	872
NHL 11	−2.32	−0.17	2.64	0.07	0.30	0.48	0.15	953
DHI 1	−3.13	−1.13	1.78	0.01	0.11	0.49	0.39	862
DHI 2	−3.31	−0.12	3.44	0.02	0.23	0.58	0.18	871
DHI 3	−3.17	−1.05	3.00	0.01	0.13	0.62	0.23	868
DHI 4	−2.37	0.52	3.82	0.04	0.31	0.51	0.14	858
DHI 5	−2.14	−0.04	3.03	0.03	0.22	0.54	0.21	852
DHI 6	−2.76	−1.39	2.05	0.01	0.09	0.55	0.35	841
DHI 7	−3.21	0.09	3.70	0.02	0.26	0.57	0.15	854
DHI 8	−2.66	0.51	4.51	0.03	0.31	0.56	0.09	826

Unordered thresholds reported in bold.

**Figure 7:**
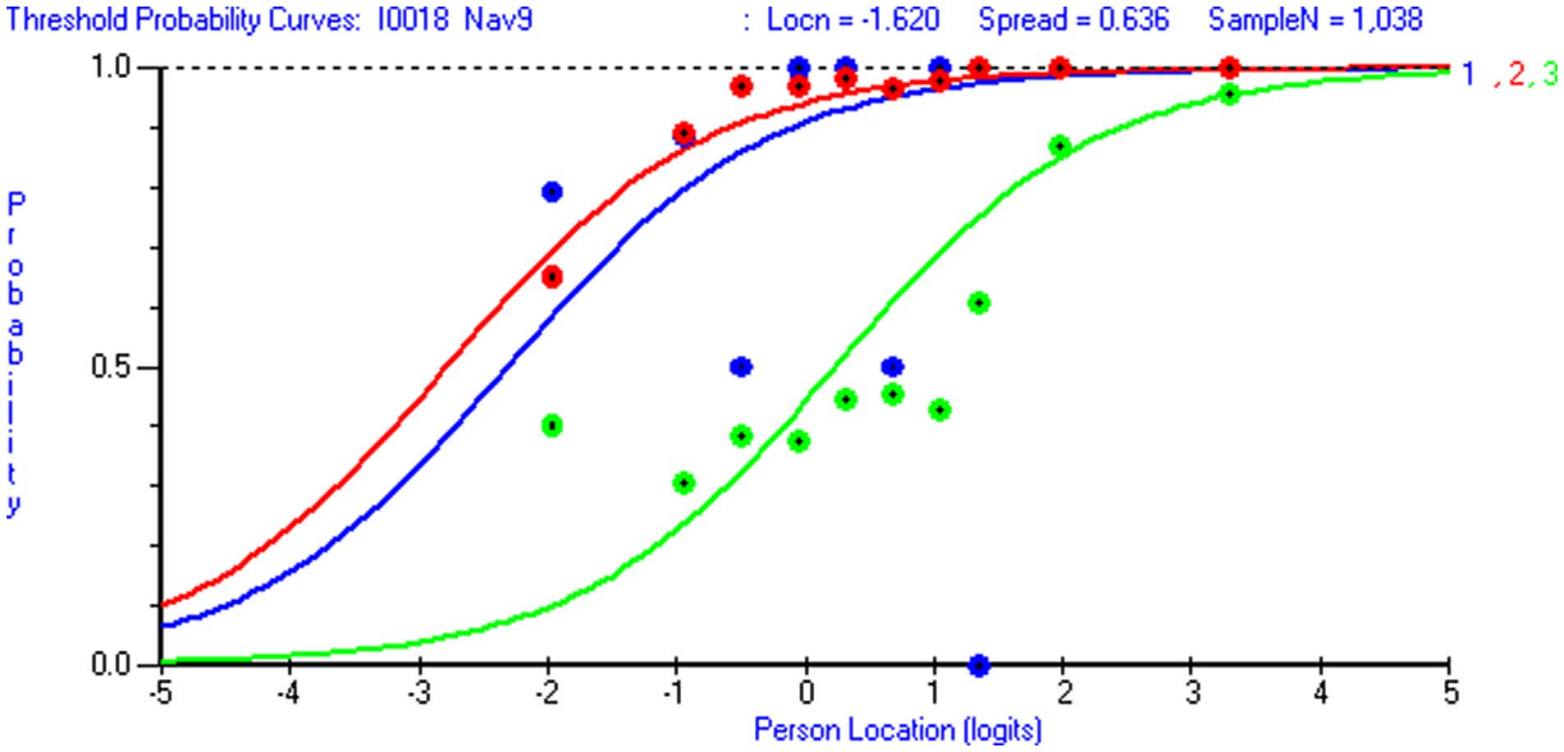
Unordered thresholds for item NHL 9. Threshold probability curves with observed data displaying unordered thresholds for item NHL 9: *Know how to book an appointment at the primary health service* (loc=-1.62; Chi Sq (p)=47.74 (.000); Infit=1.25; missing=.7 %) (generated using Rumm 2030).

Based on the findings from our evaluation, we assert that the HLS_19_-NHL and HLS_19_-DHI scales are highly relevant for assessing individuals’ utilization of healthcare services. We emphasize the importance of incorporating these assessment tools into HL population studies and studies mapping individuals with LTCs or non-communicable disease (NCDs). Furthermore, we highlight the necessity of identifying LTC or NCD populations as a distinct subgroup in future public health assessments, particularly in the context of NCD control and management. While the primary focus of this study was to evaluate the psychometric properties of the scales, no items were removed for refinement. However, considering the observed DIF and the somewhat poor targeting, we recommend adding additional items to the DHI scale to capture a broader range of digital HL levels and enhance content validity. We also suggest exploring alternative item response theory (IRT) models for latent trait regression to improve future analyses. Demographic data from our sample revealed an overrepresentation of women, underscoring their importance as a key target group within the LTC population. Additionally, with a higher proportion of individuals with LTCs residing in rural areas and the potential advantages of healthcare digitalization for long-term care (2), the inclusion of the NHL and DHI scales as essential HL assessment tools for this population is further supported. As HL is inherently a relational construct, and considering that many items in the NHL and DHI scales to a large extent refer to advanced and critical HL (86), the findings underscore the significance of assessing organizational health literacy (OHL). This involves understanding how health professionals can adapt their communication strategies to address the diverse HL levels of various patient groups, thereby ensuring the delivery of tailored, effective information. For example, items located around the middle of the scale, such as items NHL 1 and DHI 2 may reflect tasks that health personnel should be particularly attentive to when supporting patients with LTCs (Figure 7).

Against the stressed need to develop sustainable health services and improve NCD management, this study highlights the relevance of the NHL and DHI scales for assessing HL in LTC populations. The results confirm that these scales are reliable and valid for measuring navigational and digital HL in individuals with LTCs, consistent with prior validation studies (69, 87). We recommend adding items targeting higher DHI trait levels to better capture respondents with advanced digital HL capabilities. However, cross-country comparisons should account for contextual differences, particularly those related to levels of digitalization. The multidimensionality of the combined NHL and DHI scales further underscores the importance of treating these specific measures as distinct yet complimentary aspects to HL. Future research should explore the applicability of these scales in other LTC populations and validate additional HL measures for this subgroup. Moreover, examining the relationships between NHL and DHI scores, other HL measures, and sociodemographic factors will provide deeper insights into the determinants of HL. Finally, the development of a short-form HL assessment tailored to the LTC population, including well-discriminating items from various HL domains such as digital, navigational, general, vaccination-related, and communication HL, should be considered for future assessments.

Assessment scales of specific HLs, like NHL and DHI, are not grounded in the conceptual framework of comprehensive or general HL. Grounded in public health and designed to conceptualize comprehensive HL for population-level assessment, the HL framework (34) underpinning our study has harnessed critique for being too limited to the medical realm in its design and failing to address the emphasized need for health promotion (27). Population assessments of HL like HLS_19_ where demography and determinants are included, may however meet the call for moving beyond the medical view to a broader societal view expressed in public health research (27). Still, there are studies indicating associations between low levels of HL and challenges in handling necessary information and support to efficiently navigate the health care services (12), and the relevance of HL both for self-management of LTCs and for sustainable health care (17). The LTC population seems to be vital to prioritize when mapping HL in populations yet have been paid scarce attention in the field of HL research (11, 17, 18). Further testing the validity of HL scales and their applicability to adults with LTCs is part of progressing ongoing work on mapping HL in populations, to investigate the influence of demography and social determinants, and for further tracking the progress towards increased HL and quality of life (11, 22, 23, 35).

### 4.3 Strengths and limitations

Large-scale surveys provide valuable opportunities to examine the psychometric properties of measurement scales by using item response theory (IRT). To the best of our knowledge, this study represents the first effort to evaluate the psychometric properties of the Norwegian NHL scale in the LTC population and the first to assess the DHI scale in any LTC population across diagnosis and demography. Furthermore, this is the first study to present NHL and DHL data specifically from the Norwegian LTC population. The dataset and sample used in this study possess notable strengths as well as limitations. A major strength lies in the heterogeneity of the sample, which introduces a health-promoting perspective of the study of LTC, contrasting with the traditionally disease-focused clinical approach (88). The sample was derived from a population-based survey, featuring a large and representative population. This design provides detailed data, a high response rate, and minimizes the risk of selection bias. Another advantage is the inclusion of demographic data within the survey, which allows for a more comprehensive examination of the population. However, the representativeness of the LTCs sample is limited, as the respondents were included solely based on self-reported LTCs and required to have at least responded to one item in each scale. The lower number of participants responding to the DHI scale compared with the NHL is not caused by the design structure. Specifically, filter-based excluded respondents who reported not using the internet to search for health information. While such routing may affect precision when comparing groups, missing responses due to survey design are unlikely to introduce DIF, given sample-dependent item calibration (46). We underscore that the DHI results only apply for internet users.

As outlined in Table 5, a substantial number of respondents skipped item NHL 10 (*finding out how patient and user organizations or similar can help you navigate the health care system*), suggesting that the wording of this item may require refinement. Given the important role of such organizations in the health information landscape and in supporting self-management, the high rate of non-responses may indicate limited awareness of, or access to, user organizations. Since these organizations are often linked to diagnosis-specific conditions, individuals in this target population, many of whom may not be users of specialized healthcare, might encounter difficulties accessing such information. As highlighted in recent research, navigating siloed healthcare systems can itself constitute as a barrier for users (42). Considering the relevance of this task to individuals with LTCs (*finding out how patient and user organizations or similar can help you navigate the health care system*), further examination of the wording of item NHL 10 is therefore warranted. Despite these challenges, item NHL 10 discriminates well and appears out as a highly relevant item when assessing individuals with LTCs. Higher rates of missing responses were also observed for item NHL 3, which pertains to health insurance. This may reflect the structure of the Norwegian healthcare system, where health insurance plays a relatively limited role. Further examination of the wording of this item may therefore help improve its content validity and alignment also with the Norwegian context.

Additionally, a limitation to the study data stems from the self-reported nature of the data, as individuals who report having LTCs are likely to experience the impact of their illnesses in daily life (89). Those who completed the comprehensive survey may represent a subgroup of individuals who are both more affected by HL in terms of needing health information and services to manage LTCs and may likely possess a relatively higher level of HL by simply being able to be responding to such a comprehensive questionnaire. Conversely, individuals with lower levels of HL might be underrepresented. Despite this, the study’s primary goal, to assess the properties of the measurement scales and identify potential biases through misfit, addresses this limitation. We advocate for continuous sampling of the LTC and NCD populations through population-based HL assessments, both within and across countries. Using these measures in mapping the LTC population is recommended, as they are highly relevant to this target group and yield valid and reliable scales. Finally, we stress the importance of conducting further HL assessments in the LTC population, aligning with the growing call for a more person-cantered approach to LTC, in contrast to the traditional disease-oriented focus (2).

## 5 Conclusion

The scales demonstrated acceptable unidimensionality and reliability for measuring navigational health literacy (NHL) and digital health literacy (DHI) in the targeted population of individuals with long-term conditions (LTCs), supporting the estimation of one overall score for population assessment through cross-sectional and longitudinal surveying. Accordingly, our results suggest that both NHL and DHI provide sufficient measurement validity for the LTC population. However, we detected differential item functioning (DIF) indicating invariance in both scales, and item-level fit to the Rasch partial credit model (PCM) for the NHL scale was suboptimal. Consequently, alternative item response theory (IRT) models may describe the observed data better. Despite the observed deviation from the expected pattern, its magnitude was not considered sufficient to compromise the validity of the total scores. We recommend revising and further evaluating item NHL 9 on the NHL scale. Moreover, given the poor targeting observed for the DHI, additional items should be developed to yield more nuanced knowledge of population levels of digital health literacy and to strengthen construct validity of the DHI measurement scale.

## Data Availability

Publicly available datasets were analyzed in this study. This data can be found at: the dataset supporting the conclusions of this article is available in the repository of the National Study Team in Norway for HLS19, but restrictions apply to the availability of these data, which were used under license for the current study, and so are not publicly available. Data are however available from the authors upon reasonable request and with permission of National Study Team in Norway for HLS_19_.

## Glossary

AIC: Akaike Information Criterion
CATI: Computer assisted telephone interviews
CI: Class Intervals
CI: Confidence Intervals
CFI: Comparative Fit Index
DHI: The Norwegian instrument for measuring Digital Health Literacy within the dimension of digital health information
DIF: differential item functioning
GOFIs: Goodness-Of-Fit Indices
HL: Health Literacy
HLS19: International Health Literacy Population Survey Questionnaire
ISCED: The International Standard Classification of Education
LD: Local Dependence
LTCs: Long-Term Conditions
MNSQ: Mean Squared
M-POHL: WHO Action Network on Measuring Population and Organizational Health Literacy
NCDs: Non-Communicable Diseases
NHL: HLS19-NHL-NO_Norwegian – The Norwegian instrument for measuring Navigation Health Literacy
OHL: Organizational Health Literacy
PCA: Principal Component Analysis
PCM: Partial Credit Model
PF: Person Factor
PSI: Person Separation Index
RMSEA: Root Mean-Square Error of Approximation
SRMSR: Standard Root Mean Square Residual
TLI: Tucker Lewis Index
WHO: The World Health Organization
